# Bacterial Profile of External Ocular Infections, Its Associated Factors, and Antimicrobial Susceptibility Pattern among Patients Attending Karamara Hospital, Jigjiga, Eastern Ethiopia

**DOI:** 10.1155/2023/8961755

**Published:** 2023-03-10

**Authors:** Tigist Abebe, Zelalem Teklemariam, Tadesse Shume, Surafel Mekuria, Kedir Urgesa, Fitsum Weldegebreal

**Affiliations:** ^1^Jigjiga University, College of Medicine and Health Sciences, Department of Medical Laboratory Sciences, Jigjiga, Ethiopia; ^2^Haramaya University, College of Health and Medicine Sciences, Harar, Ethiopia

## Abstract

**Background:**

External ocular infection is a global public health problem. Frequently, bacteria cause an ocular infection that ranges from morbidity to loss of vision. The increasing bacterial resistance in ocular infections leads to the risk of treatment failure with possibly serious consequences.

**Objective:**

The study aimed to assess the bacterial profile of external ocular infections, their associated factors, and antimicrobial susceptibility pattern among patients admitted to Karamara hospital, Jigjiga, Eastern Ethiopia.

**Method:**

Institutional-basedcross-sectional study was conducted on 288 conveniently selected patients among patients admitted to Karamara hospital from May 1 to June 30, 2020. Data were collected using a structured questionnaire. The ocular sample was collected and cultured in the appropriate culture media and identified using a series of biochemical tests. Antimicrobial susceptibility testing of isolates was performed by using the disk diffusion method. Data were double entered onto EpiData version 3.1 then exported to SPSS version 20 and analyzed to calculate descriptive frequency and odds ratio, and *p* value ≤0.05 was taken as the significant value.

**Result:**

The prevalence of bacterial infection in external ocular samples was 62.2% (95% CI: 56.6%, 68.4%). Out of the 179 isolates, the majority of the bacterial isolates (87.7%) were Gram-positive. *Staphylococcus aureus* (53.1%) was the predominant isolate. Using soap for washing the face (AOR = 0.43; 95% CI: 0.29, 0.95), having diabetes mellitus (AOR = 3.11; 95% CI: 1.45, 6.75), and history of hospitalization (AOR = 2.82; 95% CI: 1.44, 5.54) were significantly associated with external ocular infection. Most (95.5%) of the Gram-positive bacteria showed resistance to penicillin, but they were susceptible to vancomycin, clindamycin, and ciprofloxacin.

**Conclusion:**

The study showed a high prevalence of bacterial infections with the predominant isolate was *S. aureus*. Penicillin-resistant bacteria were identified among Gram-positive bacterial isolates. Soap usage, hospitalization, and diabetes mellitus were associated with the infection. Antibiotics that were susceptible to the specific bacteria should be used as a drug of choice and using soap for washing the face is advisable to protect against external ocular infection.

## 1. Introduction

Microorganisms are closely associated with external ocular infection. Particularly, infections caused by bacteria are quite common [1]. The most common external ocular infections include conjunctivitis, blepharitis, dacryocystitis, orbital, and periorbital cellulitis. These infections are among the leading causes of ocular morbidity and blindness worldwide, chiefly in developing countries like Ethiopia [2, 3]. Despite considerable resident microbiota, the eye is exposed to an external environment where a range of microorganisms is also inhibited which can cause eye infections opportunistically [4]. Several bacteria play a great role in triggering eye infections and corneal [5, 6]. The common bacterial agents responsible for ocular infections include Gram-positive bacteria such as *Staphylococcus aureus, Staphylococcus epidermidis,* and several *Streptococcus* and *Bacillus* spp. as well as Gram-negative bacteria such as *Pseudomonas aeruginosa, Moraxella* spp., and *Haemophilus* spp [7]. These organisms may come from the patient's skin, upper respiratory tract, or caught from another person with an external ocular infection [8].

Although antibiotics have been used systemically or topically to control ocular infection, bacterial resistance has been emerging and increasing worldwide for treating ocular infections, more likely due to widespread and inappropriate dosing of broad-spectrum antibiotics for systemic infections, exacerbated by inadequate compliance to full-treatment duration [9, 10].

External ocular infections are affecting and leading to vision loss globally [11]. According to the World Health Organization (WHO), 285 million people were visually impaired worldwide. Out of those, 39 million people were blinded by the year 2010. The report also disclosed that more than 90% of the world's visually impaired people live in developing countries, and surprisingly 82% of the visual impairment, including blindness, was preventable [12]. In Africa, it is estimated that approximately 2.2 million people were blinded due to ocular infection [13]. One report (2015) in Sudan showed that bacterial external ocular infections are significantly prevalent among the pediatrics population and cause more than 65% of morbidity in all cases [14].

A report in 2015 showed that, blindness in Ethiopia reached 1.6%. Out of this, 87.4% of blindness was a result of a bacterial pathogen [15]. The morbidity of ocular infections occurs ranging from mild and self-limiting conditions to extremely serious and visually threatening [16]conditions. People who are living in a rural area, children and old aged people are the most affected group compared to others [17]. Several factors such as personal hygiene, living condition, sociodemographic or economic status, ocular trauma, frequency of face washing, the occurrence of systemic disease, and cigarette smoking were considered as associate factors for bacterial-based ocular infections [7, 15].

Most ocular infections in the world have been treated using commonly known antimicrobials. Due to this, microbial resistance to antimicrobial agents has become increasingly prevalent in ocular infections including systemic infections on a global basis [18, 19]. Particularly, in Ethiopia, there is an inordinate habit of using different antibiotics without prescription [9, 10] and there are poor personal hygiene and infection control practices, which lead to increased antimicrobial resistance in the community [20].

In Ethiopia, there are inadequate published resources on this topic. Moreover, in the study area, there is no single study conducted related to this topic. Therefore, this study was designed to determine the bacterial profile of external ocular infections, their associated risk factors, and antimicrobial susceptibility pattern among patients admitted to Karamara hospital Jigjiga, Eastern Ethiopia.

## 2. Methods and Materials

### 2.1. Study Area and Period

The study was conducted in Karamara hospital in Jigjiga from May 1 to June 30, 2020. Jigjiga town is found in the eastern part of Ethiopia, and it is the capital city of the Somali region. It is found 635 km away from Addis Ababa. Karamara hospital renders health services for over seven million people living in all zones and districts of the Somali region. It has high patient flow in the eye clinic.

### 2.2. Study Design and Population

An institutional-basedcross-sectional study was employed. Two hundred eighty-eight (288) patients who visited the eye clinic of Karamara hospital with suspected external ocular infections and fulfilled the inclusion criteria during the study period were included. Patients on antibiotics, anti-inflammatory drugs, and those diagnosed with allergic problems and trachoma were excluded.

### 2.3. Sample Size Determination and Sampling Techniques

The sample size of the study was determined using a single population proportion formula by considering the prevalence of bacterial pathogens among patients with external ocular infection (21%) from the study conducted in Hawassa University Teaching and Referral Hospital, southern Ethiopia [15], with 95% confidence interval (CI), 5% margin of error and 10% nonresponse rate. Then, the final sample size was 288. The study participants were recruited conveniently until we got the required sample size.

### 2.4. Data Collection Methods

#### 2.4.1. Physical Examination, Specimen Collection, and Transportation

All patients suspected with external ocular infections were physically examined using a slit-lamp biomicroscope and diagnosed by an ophthalmologist. Specimens from the external part of the eye, such as conjunctiva, eyelid, and lacrimal sac, were taken by an ophthalmologist. Conjunctival specimens were collected using a sterile saline moistened cotton swab, applied by passing the swab gently over the lower and upper conjunctiva 2-times [21]. In cases of dacryocystitis, specimens were taken by puncture and aspiration of the lacrimal sac. An antiseptic was first applied to the area of the puncture, and then the lacrimal sac was punctured in the area below the medial canthal ligament [22]. In the case of blepharitis, discharge from the margin of the eyelid was collected using cotton swabs and placed into a sterile tube. All the swabs were finally immersed in a tube that had 3 ml brain heart infusion (BHI) [23] and transported to the Somali regional microbiology laboratory by using the cold box. After specimen collection, data on sociodemographic and associated factors with external ocular infection were collected by a trained optometrist from each study participant using a pretested structured questionnaire adapted from the previous studies [15, 24].

### 2.5. Bacterial Isolation and Identification

Gram staining was done for differentiating Gram-positive and Gram-negative bacteria and to observe the presence and morphology of cells. Smears were prepared at the collection sites from swabs by gently circularly spreading the specimen on a glass slide [25]. Each specimen was inoculated on a blood agar plate (BAP), chocolate agar plate (CAP), MacConkey agar (MAC), and mannitol salt ager (MSA) culture media with sterile wire loops and incubated at 37°C for 48 hours. Chocolate agar plates were incubated within a candle-jar to facilitate the CO_2_ atmosphere. After 24 hours of incubation, the plates were observed and examined for bacterial pathogen growth, and plates with no growth were reincubated for further 24 hours [26].

The identification of bacterial pathogens was done initially by Gram stain and colony morphology from culture followed by biochemical tests. Biochemical tests like catalase, coagulase, optochin disk, and bile solubility tests were applied to identify and differentiate Gram-positive cocci, while biochemical tests, such as triple sugar iron agar (TSI), citrate utilization, lysine decarboxylase agar (LDC), oxidase, urease, indole, Methyl Red-Voges-Proskauer (MR-VP), and tributyrin tests were used to identify Gram-negative bacterial isolates [26, 27]. Gram-positive bacteria were identified using hemolytic activity on sheep blood agar, catalase for differentiation of Gram-positive and Gram-negative, coagulase test for *S. aureus*, bile solubility, and optochin disk test sensitivity for *S. pneumonia* [26].

### 2.6. Antimicrobial Susceptibility Testing

An antimicrobial susceptibility testing was carried out on each identified bacterium using the disc diffusion method on Mueller Hinton agar (MHA). Besides, MHA medium containing 5% defibrinated sheep blood was used for fastidious bacterial isolate like *S. pneumoniae*. Primarily, 3–5 bacterial colonies of the test organism were picked and emulsified in 5 ml of nutrient broth and mixed gently. To standardize the density of the inoculum for a susceptibility test, a 0.5 McFarland standard solution was used. The plates were then inoculated by streaking the swab over the entire agar surface then the common antimicrobials were used for patients treated in the Karamara general hospital with the following concentrations: ceftriaxone (CRO) (30 *μ*g), ciprofloxacin (CIP) (5 *μ*g), trimethoprim-sulfamethoxazole (SXT) (25 *μ*g), gentamicin (CN) (10 *μ*g), tetracycline (TE) (30 *μ*g), penicillin (P) (10U) and clindamycin (DA) (2 *μ*g), vancomycin (VA) (30 *μ*g), and doxycycline (DO) (30 *μ*g) were placed using sterile forceps on the plate's surface on the MHA plate and incubated at 37°C for 18–24 hours but for *S. aureus,* it was incubated for only 16–18 hours, and then the zone of inhibition was determined. The zone diameters were measured and recorded. Finally, bacterial susceptibilities were interpreted following the Clinical and Laboratory Standards Institute (CLSI) guidelines as susceptible (S), intermediate (I), or resistant [28].

### 2.7. Quality Control

The reliability of this study was ensured by actualizing quality control (QC) measures all through the entire process of the laboratory procedures. All necessary materials, equipment, and procedures were controlled enough. The questionnaire was prepared in English language and translated to Amharic and the Somali language then retranslated to English to check the consistency. The data were collected by a trained optometrist. Needed specimens were collected following the standard operating procedures (SOPs) that were prepared specifically for external ocular specimen collection. Culture media sterility was ensured by incubating uninoculated media. The prepared culture media performance was checked by inoculating the standard strains, such as *Escherichia coli* (ATCC 25922), *Staphylococcus aureus* (ATCC 25923), and *Pseudomonas aeruginosa* (ATCC 27853) [25, 27] obtained from the Ethiopian Public Health Institute, Addis Ababa, Ethiopia. These strains were also used to check the qualities of biochemical tests. Furthermore, the quality of the data entry was maintained by double data entry.

### 2.8. Data Analysis

The data were cleaned, coded, and double entered using EpiData version 3.1 software and then exported to statistical package for Social Sciences version 20 software for analysis. The descriptive statistics (mean, percentages or frequency) were calculated to summarize the findings. The magnitude of the association between the different variables to the outcome variables was measured by the odds ratio with a 95% confidence interval (CI). Bivariate and multivariable logistic regression analyses were performed to assess the association between dependent and independent variables. Crude odds ratio (COR) and adjusted odds ratio (AOR) at 95% confidence interval was used to measure the strength of association. Those variables with *p* value <0.2 at bivariate logistic regression were considered for the multivariable logistic regression model to control the confiding variables. Statistical significance was declared at a *p* value less than 0.05.

### 2.9. Ethical Consideration

Ethical clearance was obtained from the Institutional Health Research Ethics Review Committee (IHRERC) Health and Medical Sciences College, Haramaya University. An official permission letter was written to the Somali regional health office which wrote a permission letter to Karamara hospital. The objective, purpose, risk, and benefits were explained and the signed consent was obtained from the hospital head, the study participants and guardian, or parents of children under 18 years. All the information obtained from the study participants were kept confidential but positive culture result with the possible drug of choice was reported to the ophthalmologist for proper treatment.

## 3. Result

### 3.1. Sociodemographic Characteristics

A total of 288 patients were clinically diagnosed with external ocular infections and included in this study with a response rate of 100%. About 52.8% of the study participants were males. The mean age was 38.5 (SD ± 16.2) years and 49% of the study participants were of the age between 18 and 39 years. Approximately 71.2% of the participants were from urban and 31.3% were businessmen. One-fourth of the study participants had formal education up to the primary school level (Table 1).

### 3.2. Behavioral and Clinical Related Factors

This result showed that among the participants, 48 (16.7%) had swimming habits, 112 (38.9%) of them frequently washed their faces, 176 (61%) used soap for washing their faces, and 80 (27.8%) used eye cosmetics. Patients who had a previous history of ocular surface disease were 70 (24.3%), hospitalized 78 (27%), ocular trauma 35 (12%), using contact lens 10 (3.5%) making eye surgery 43 (14.9%), and having diabetes were 56 (19.4%) (Table 2).

### 3.3. Bacterial Profile

Among 288 ocular specimens subjected to culture, 62.2% (179/288) (95% CI: 56.6%, 68.4%) were positive for different bacterial species. Among the 179 isolates, 87.7% were Gram-positive cocci with a predominant isolate of *S. aureus* (53.1%), followed by Coagulase-negative staphylococcus (932.4%), and *Streptococcus pneumonia* (2.2%). However, 12.3% of isolates were Gram-negative bacteria with predominantly *E. coli* spp (6.2%). In addition, other species were less frequent such as *Klebsiella-pneumonia* (3.3%) were isolated only in the case of conjunctivitis, and *Pseudomonas aeruginosa* (1.7%) was isolated in the case of both conjunctivitis and blepharitis (Table 3).

### 3.4. The Magnitude of External Ocular Infection

The prevalence of conjunctivitis was 48.3% (95% CI: 42.7%, 54.5%) followed by blepharitis (29.9%) (95% CI: 24.3%, 35.1%), blepharoconjunctivitis (14.6%) (95% CI: 10.4%, 18.7%), and dacrocystitis 7.3% (95% CI: 4.5%, 10.4%). In this study*, S. aureus* (55.8% in conjunctivitis, 50% in blepharitis, 40% blepheroconjunctivitis, and 50% dacryocystitis) was the most dominant amongst the other strains.

### 3.5. Associated Factors

In this study, washing with soap, educational status, presence of ocular surface disease, diabetes mellitus, hospitalization, and making surgery showed a significant association in bivariate logistic regression analysis (*p* < 0.25) and were considered as a candidate for multivariable logistic regression. In multivariable analysis, washing with soap, hospitalization, and presence of diabetes mellitus was statistically significant in patients who had an external ocular infection at *p* value less than 0.05.

Patients who used soap for washing their faces were 56.7% (AOR = 0.43; 95% CI: 0.29, 0.95) less likely to be infected with bacterial external ocular infection compared to counterparts. Patients who had a history of hospitalization up to 30 days were 2.8 times (AOR = 2.82; 95% CI: 1.44, 5.54) more likely to develop an external ocular infection compared to their counterparts. Patients who were diabetic were 3.11 times (AOR = 3.11; 95% CI: 1.45, 6.75) more likely to develop the infection than those who did not have diabetes (Table 4).

### 3.6. Antimicrobial Susceptibility Patterns of Gram-Positive Bacterial Isolate

The antimicrobial susceptibility pattern of bacteria was done on nine antimicrobial agents. Out of those, vancomycin, penicillin, and doxycycline were tested for Gram-positive bacteria. Most (150/95.5%) of the Gram-positive showed resistance for penicillin, but they were susceptible to vancomycin (152/96.8%), clindamycin (148/94.3%), ciprofloxacin (145/92.3%), doxycycline (132/84.1%), ceftriaxone (112/71.3%), gentamicin (104/66.2%), tetracycline (88/56.0%), and trimethoprim-sulfamethoxazole (76/48.4%) (Table 5).

### 3.7. Antimicrobial Susceptibility Patterns of Gram-Negative Bacterial Isolate

Over 68% and 63% of Gram-negative bacteria isolates were sensitive to gentamicin and ceftriaxone, respectively. However, 50% and 77.3% were resistant to tetracycline and trimethoprim-sulfamethoxazole, respectively (Table 6).

### 3.8. Multi-Drug Resistance

In this study, the overall multidrug resistance (resistance to two or more antimicrobials) was 87.7%. Only 2.2% were sensitive to all tested antimicrobials (Table 7).

## 4. Discussion

External ocular infection is one of the major problems affecting many individuals and is responsible for the increased incidence of morbidity and blindness globally [9, 29]. In the present study, the overall prevalence of bacterial pathogens was 62.2%. This is comparable with the reports which were done in Gondar (58.3%) [24], Dessie (59.4%) [15], Gondar (60.8%) [30] in Ethiopia, Sudan (63.7%) [14], and Uganda (59.5%) [31]. On the other hand, it is lower compared to reports from Jimma, Southwest Ethiopia (74.7%) [6], Nigeria (81.7%) [32], and India (88%) [33]. However, it is higher from the study conducted in Hawassa, Ethiopia (48.8%) [34]. This variation might be due to the difference in geographical variation, time variation, study population, and the practice of infection control in the community.

In this study, Gram-positive cocci (87.7%) were the most common isolates. This is supported by previous studies conducted in Gondar (88%) [24], Dessie (55.6%) [15], and Jimma (52%) [6] in Ethiopia, and Nigeria (50.3%) [35]. In the current study, the predominant bacterial isolates were *S. aureus* (53.1%) similar to other previous studies conducted in Gondar [24], Jimma [6], Nigeria [35], and India [1]. Other studies reported that CoNS was the predominant isolate [15]. The occurrence of different bacteria as an etiological agent for external ocular infection may be due to differences in the environmental conditions [36].

The current study showed a higher prevalence of conjunctivitis (58%) and blepharitis as the next most dominant types of eye infection (33.5%). This is consistent with a study conducted in northwest Ethiopia [15]. *Staphylococcus aureus* was the most common isolate in conjunctivitis (55.8%), blepharitis (50%), and blepharoconjunctivitis (40%). A similar conclusion was reached by studies conducted in Ethiopia [6] and India [33]. On the other hand, *S. aureus* was isolated from blepharitis (47.6%) and conjunctivitis (26.6%) as reported from northern Ethiopia [9]. This dominance of *S. aureus* might be due to contamination of the eye from skin normal flora as a result of touching the eyes with contaminated hands [37].

In the present study, those who used soap were less likely to develop an external ocular infection. It increases personal hygiene, which prevents the growth of bacterial pathogens on the exterior part of the eye, and it is supported by a similar study conducted in France [38]. However, another study reported that there is no significant association between soap usage and external ocular infection [15]. This protective association might be due to the chemical characteristics of soap, which destroys the pathogen from the infection site.

History of hospitalization was significantly associated with external ocular infection. This is consistent with the study conducted in Portugal [39] and in the USA, Central California [40]. The main reason for this significant association is due to the characteristics of the bacteria that cause external ocular infections. These bacteria cause nosocomial infection, that can be acquired during hospitalization [41].

Being diabetes mellitus was significantly associated with ocular infection, this result is supported by several studies in China [42], Denmark [43], England [44], and Iran [3]. This is due to individuals with diabetes mellitus having lower immunity, which may result in loss of control for systemic infections with subsequent spread to ocular tissues [43].

The drug susceptibility patterns of Gram-positive cocci bacterial isolates showed that sensitivity to vancomycin (96.8%) followed by ciprofloxacin (92.4%). This finding agrees with studies conducted in Ethiopia [6] and India [33]. However, most of the isolates were resistant to penicillin and a similar pattern of results was obtained in Jimma [6] and Gondar [24]. This reduction in the effectiveness of penicillin could be due to the frequent usage that results from its low price and accessibility without a prescription.

Most of the Gram-negative isolates were sensitive to gentamicin (68%) followed by ceftriaxone (63%), but they were resistant to trimethoprim-sulphamethoxazole (78%). Several reports also showed similar patterns of drug resistance among Gram-negative bacteria Dessie, [15] Gondar, [20], and India [2]. *Moraxella species* were 100% sensitive to ciprofloxacin, gentamicin, and tetracycline. This might be due to the few numbers of isolated *Moraxella species.* Their sensitivity to ciprofloxacin is consistent with the studies conducted in Jimma [6] and Hawassa [45]. However, they showed 100% resistance to trimethoprim-sulphamethoxazole. This might be due to a few isolates of the species.

In this study, most bacterial isolates were resistant to penicillin. This might be due to the usage of those broad-spectrum antimicrobial agents without taking appropriate diagnosis. This result is supported by the study conducted in Jimma [6].

The prevalence of multidrug resistance (MDR) to two or more bacterial isolates to the commonly prescribed antimicrobials was observed in 87.7% of the isolates. This is consistent with what has been found in previous studies conducted in Gondar, northwest Ethiopia [18]. However, a lower prevalence of multidrug resistance was previously reported in Hawassa, south Ethiopia [34]. This may be due to the difference in type and generation of antibiotics that we used for susceptibility testing.

## 5. Conclusion

In this study bacterial external ocular infections are highly prevalent. Conjunctivitis was the dominant external eye infection followed by blepharitis. Gram-positive bacteria constitute more than eighty-five percent of isolates with *S. aureus* being the most predominant ones. Vancomycin and clindamycin were the drugs of choice for Gram-positive bacterial isolate and gentamicin and ceftriaxone were the drugs for a Gram-negative bacterial isolate. The prevalence of MDR to the commonly prescribed antimicrobials was very high. In this study, soap usage, hospitalization, and diabetes mellitus were statistically significant. Therefore, the community should keep themselves from systemic diseases like DM and practice good personal hygiene to minimize the probability of getting external ocular infections. Antibiotics that have high sensitivity for each bacterial isolate should be used as a drug of choice for patients with external ocular infection. Using soap for washing the face is advisable to protect against external ocular infection. Additionally, Somali regional health offices should give health education to the community to minimize the effect of possible risk factors.

## Figures and Tables

**Table 1:** Sociodemographic characteristics of the study participants with external ocular infections attending at Karamara hospital, Jigjiga, eastern Ethiopia, 2020.

Variables		Number (percentage) *N* (%)
Residence	Rural	83 (28.8)
	Urban	205 (71.2)

Sex	Male	152 (52.8)
	Female	136 (47.2)

Age group in year	0–11	9 (3.1)
	12–17	17 (5.9)
	18–39	141 (49)
	≥40	121 (42)

Ethnicity	Somali	135 (46.9)
	Amhara	39 (13.5)
	Oromo	51 (17.7)
	Gurage	18 (6.3)
	Tigray	11 (3.8)
	Wolayta	25 (8.7)
	Others	9 (3.1)

Marital status	Underage	25 (8.7)
	Single	63 (21.9)
	Married	154 (53.5)
	Divorced	19 (6.6)
	Widowed	27 (9.4)

Employment	Student	44 (15.3)
	Housewife	51 (17.7)
	Civil servant	52 (18.1)
	Farmer	30 (10.4)
	Businessman	90 (31.3)
	Others	21 (7.3)

Educational status	Not read and write	44 (15.3)
	Read and write	57 (19.8)
	Primary school	62 (21.5)
	High school	72 (25.0)
	College and above	53 (18.4)

Family income	<1000	19 (6.6)
	1100–3000	158 (54.9)
	3100–6000	90 (31.3)
	>6000	21 (7.3)

**Table 2:** Personal and clinical related factors of patients attending at Karamara hospital, Jigjiga, eastern Ethiopia, 2020.

Variables		Frequency (%)
Swimming habit	Yes	48 (16.7)
	No	240 (83.3)

Frequency of face wash	Once per day	116 (40.3)
	Twice per day	55 (19.1)
	Three times per day	112 (38.9)
	>four times per day	5 (1.7)

Usage of soap	Yes	176 (61)
	No	112 (39)

Usage of eye cosmetics	Yes	80 (27.8)
	No	208 (72.2)

Have ocular surface disease	Yes	70 (24.3)
	No	218 (75.7)

Hospitalization	Yes	78 (27)
	No	210 (73)

Have ocular trauma	Yes	35 (12)
	No	253 (88)

Usage of contact lens	Yes	10 (3.5)
	No	278 (96.5)

Have diabetic mellitus	Yes	56 (19.4)
	No	232 (80.6)

Making eye surgery	Yes	43 (14.9)
	No	245 (85.1)

**Table 3:** Bacterial profiles among different clinical features of external ocular infection attending at Karamara hospital, Jigjiga, eastern Ethiopia, 2020.

Bacterial isolate	Conjunctivitis *N* = 139	Blepharitis *N* = 86	Blepharo-conjunctivitis*N* = 42	Dacryocystitis *N* = 21	Total *N* = 179
Gram-positive cocci *Staphylococcus aureus*	58 (55.8%)	30 (50%)	2 (40%)	5 (50%)	95 (53.1%)
*Streptococcus pneumonia*	2 (1.9%)	2 (3.3%)	—	—	4 (2.2%)
*Coagulase-negative staphylococcus*	32 (30.8%)	22 (36.7%)	1 (20%)	3 (30%)	58 (32.4%)
Gram-negative cocci *Moraxella* spp	—	—	1 (20%)	1(10%)	2 (1.1%)
Gram-negative bacilli *Pseudomonas-aeruginosa*	1 (1%)	2 (3.3%)	—	—	3 (1.7%)
*Escherichia. coli*	5 (4.8%)	4 (6.7%)	1 (20%)	1 (10%)	11 (6.2%)
*Klebsiella-pneumonia*	6 (5.7%)	—	—	—	6 (3.3%)

**Table 4:** Bivariate and multivariable analysis of bacterial infection of the eye among external ocular infections at Karamara hospital, Jigjiga, Ethiopia, 2020.

Variables	Bacterial isolates No (%)		COR (95% CI)	*p*-value	AOR (CI 95%)	*p*-value
	Yes	No				
Washing with soap						
Yes	97 (55.1)	79 (44.9)	0.45 (0.27, 0.75)	0.002	0.43 (0.30, 0.95)	**0.034** ^*∗*^
No	82 (73.2)	30 (26.8)	1			
Educational status						
Not read and write	10 (22.7)	34 (77.3)	2.81 (1.16, 6.84)	0.02	0.61 (0.23, 1.65)	0.33
Read and write	19 (33.3)	38 (66.7)	1.65 (0.76, 3.58)	0.20	0.80 (0.34, 1.87)	0.61
Primary school	18 (29)	44 (71)	2.02 (0.94, 4.37)	0.07	0.59 (0.26, 1.34)	0.21
High school	38 (52.8)	34 (47.2)	0.74 (0.36, 1.51)	0.41	1.34 (0.62, 2.85)	0.46
College and above	24 (45.3)	29 (54.7)	1		1	
Ocular surface disease						
Yes	53 (75.7)	17 (24.3)	2.28 (1.24, 4.18)	0.008	1.74 (0.88, 3.44)	0.11
No	126 (57.8)	92 (42.2)	1			
Hospitalization						
Yes	63 (80.8)	15 (19.2)	3.40 (1.82, 6.36)	0.001	2.82 (1.44, 5.53)	**0.003** ^*∗*^
No	116 (55.2)	94 (44.8)	1			
DM						
Yes	46 (82.1)	10 (17.9)	3.42 (1.65, 7.12)	0.001	3.11 (1.44, 6.75)	**0.004** ^*∗*^
No	133 (57.3)	99 (42.7)	1			
Making surgery						
Yes	34 (79.1)	9 (20.9)	2.60 (1.20, 5.67)	0.016	1.57(0.67, 3.66)	0.30
No	145 (59.2)	100 (40.8)	1			

^*∗*^ = statistically significant.

**Table 5:** Antimicrobial susceptibility pattern of gram-positive bacteria isolated from external ocular infection at Karamara hospital, Jigjiga, eastern Ethiopia, 2020.

Organisms isolated *N* = 157		Antibiotics								
		VAN	PE	DO	CIP	CN	DA	SXT	TE	CRO
*Staphylococcus-aureus n* = 95	S	90 (94.7%)	4 (4.2%)	72 (75.8%)	87 (91.6%)	83 (87.4%)	91 (95.8%)	45 (47.4%)	46 (48.4%)	70 (73.7%)
	I	1 (1.1%)	—	5 (5.3%)	2 (2.1%)	—	—	5 (5.2%)	23 (24.2%)	—
	R	4 (4.2%)	91 (95.8%)	18 (18.9%)	6 (6.3%)	12 (12.6%)	4 (4.2%)	45 (47.4%)	26 (27.4%)	25 (26.3%)

*Coagulase-negative-staphylococcus n* = 58	S	58 (100%)	—	56 (96.6%)	55 (94.8%)	19 (32.8%)	54 (93%)	30 (51.7%)	39 (67.2%)	40 (69%)
	I	—	—	—	—	1 (1.7%)	—	—	—	2 (3.4%)
	R	—	58 (100%)	2 (3.4%)	3 (5.2%)	38 (65.5%)	4 (7%)	28 (48.3)	19 (32.8%)	16 (27.6%)

*Streptococcus-pneumonia n* = 4	S	4 (100%)	3 (75%)	4 (100%)	3 (75%)	2 (50%)	3 (75%)	1 (25%)	3 (75%)	2 (50%)
	I	—	—	—	—	—	—	—	—	1 (25%)
	R	—	1 (25%)	—	1 (25%)	2 (50%)	1 (25%)	3 (75%)	1 (25%)	1 (25%)

CoNS: *Coagulase-negative staphylococci*, VAN-vancomycin, PE-penicillin, DO-doxycycline, CIP-ciprofloxacin, CN-gentamicin, DA-clindamycin, TE-tetracycline, SXT-trimethoprim-sulfamethoxazole, CRO-ceftriaxone, S sensitive, I intermediate, R resist.

**Table 6:** Antimicrobial susceptibility pattern of gram-negative bacteria isolated from external ocular infection at Karamara hospital, Jigjiga, eastern Ethiopia, 2020.

Organisms isolated *n* = 22		Antibiotics				
		CIP	CN	SXT	TE	CRO
*Moraxella* spp *n* = 2	S	2 (100%)	2 (100%)	—	2 (100%)	1 (50%)
	I	—	—	—	—	—
	R	—	—	2 (100%)	—	1 (50%)

*Pseudomonas-aeruginosa n* = 3	S	3 (100%)	2 (66.7%)	—	—	2 (66.7%)
	I	—	1 (33.3%)	—	—	—
	R	—	—	3 (100%)	3 (100%)	1 (33.3%)

*Escherichia-coli n* = 11	S	3 (27.3%)	5 (45.5%)	1 (9%)	2 (18.2%)	5 (45.5%)
	I	—	1(9%)	—	2 (18.2%)	—
	R	8 (72.7%)	5 (45.5%)	10 (91%)	7 (63.6%)	6 (54.5%)

*Klebsiella-pneumonia n* = 6	S	3 (50%)	6 (100%)	4 (66.7%)	5 (83.3%)	6 (100%)
	I	3 (50%)	—	—	—	—
	R	—	—	7 (33.3%)	1 (16.7%)	—

CoNS: *Coagulase-negative staphylococci*, VAN-vancomycin, PE-penicillin, DO-doxycycline, CIP-ciprofloxacin, CN-gentamicin, DA-clindamycin, TE-tetracycline, SXT-trimethoprim-sulfamethoxazole, CRO-ceftriaxone. S sensitive, I intermediate, R resistance.

**Table 7:** Multidrug resistance pattern for patients with external ocular infection attending at Karamara hospital, Jigjiga, eastern Ethiopia, 2020.

Organisms	Antibiotic pattern						
	R0	R1	R2	R3	R4	>R5	Total (%)
*Staphylococcus aureus*	—	14	39	32	7	3	95 (53.1)
*Coagulase-negative-staphylococcus*	—	1	21	21	14	1	58 (32.4)
*Streptococcus pneumonia*	—	1	1	1	1	—	4 (2.2)
*Moraxella* spp	—	1	1	—	—	—	2 (1.1)
Pseudomonas *aeruginosa*	—	—	2	1	—	—	3 (1.7)
*Escherichia. coli*	1	—	1	4	3	2	11 (6.2)
*Klebsiella pneumonia*	3	1	1	1	—	—	6 (3.3)
Total (%)	4 (2.2)	18 (10)	66 (36.9)	60 (33.5)	25 (14)	6 (3.4)	179 (100)

R0: sensitive for all, R1: resistance to one drug, R2: resistance to two drugs, R3: resistance to three drugs, R4: resistance to four drugs, ≥R5: resistance to five and above drugs.

## Data Availability

All the generated data and the analysis developed in this study are included within the article.
